# Characterization and Prevalence of *Campylobacter* spp. From Broiler Chicken Rearing Period to the Slaughtering Process in Eastern China

**DOI:** 10.3389/fvets.2020.00227

**Published:** 2020-04-30

**Authors:** Yuanyue Tang, Qidong Jiang, Haiyan Tang, Zhenyu Wang, Yi Yin, Fangzhe Ren, Linghua Kong, Xinan Jiao, Jinlin Huang

**Affiliations:** ^1^Jiangsu Key Laboratory of Zoonosis, Yangzhou University, Yangzhou, China; ^2^Key Laboratory of Prevention and Control of Biological Hazard Factors (Animal Origin) for Agrifood Safety and Quality, Ministry of Agriculture of China, Yangzhou University, Yangzhou, China; ^3^Jiangsu Co-innovation Center for Prevention and Control of Important Animal Infectious Diseases and Zoonoses, Yangzhou University, Jiangsu, China; ^4^Joint International Research Laboratory of Agriculture and Agri-Product Safety of the Ministry of Education, Yangzhou University, Jiangsu, China; ^5^Lianshui Animal Husbandry and Veterinary Station, Lianyungang, China; ^6^Department of Quality and Safety Control, Heyi Food Co. Ltd., Zaozhuang, China

**Keywords:** *Campylobacter*, broiler chicken production, *Campylobacter jejuni*, *Campylobacter coli*, whole genome sequencing

## Abstract

*Campylobacter* is one of the most important foodborne pathogens worldwide, and poultry is regarded as the main reservoir of *Campylobacter*. The contamination of *Campylobacter* in broiler chickens at the farm level is closely related to the transmission of *Campylobacter* in the poultry production chain. This study identified 464 *Campylobacter* isolates from 1,534 samples from broiler rearing period and slaughtering process including 233 *Campylobacter jejuni* isolates and 231 *Campylobacter coli* isolates. We have observed a dynamic distribution of *Campylobacter* during broiler chicken production, that 66.3% of Campylobacter isolates were *C. jejuni* during broiler rearing period, while *C. coli* occupied 60.4% of Campylobacter isolates during the broiler slaughtering process. A tag-label method allowed us to track the dynamic of *Campylobacter* in each broiler chicken from 31-day age at rearing to the partition step in the slaughterhouse. At the 31-day during rearing, 150 broiler chicken were labeled, and was tracked for Campylobacter positive from rearing period to slaughtering process. Among the labeled broiler, 11 of the tracking broiler samples were able to detect *Campylobacter* from rearing period to slaughtering. All *Campylobacter* isolates from the 11 tracking samples were sequenced and analyzed. *C. jejuni* isolates were divided into four STs and *C. coli* isolates were divided into six STs. Isolates with identical core genome were observed from the same tag-labeled samples at different stages indicating a vertical transmission of *Campylobacter* in the early broiler meat production. Meanwhile, the core genome analysis elucidated the cross-contamination of *Campylobacter* during the rearing period and the slaughtering process. The virulotyping analysis revealed that all *C. jejuni* isolates shared the same virulotypes, while *C. coli* isolates were divided into three different virulotypes. The antimicrobial resistance gene analysis demonstrated that all *Campylobacter* isolates contained at least two antibiotic resistance genes (ARGs), and the ARG profiles were well-corresponding to each ST type. Our study observed a high prevalence of *Campylobacter* during the early chicken meat production, and further studies will be needed to investigate the diversity and transmission of *Campylobacter* in the poultry production chain.

## Introduction

*Campylobacter* is a leading cause of foodborne gastroenteritis in humans with an infection dose causing *Campylobacter*iosis as low as 500–800 CFU (1, 2). Poultry is a major reservoir of *Campylobacter*, which takes the main response to transmit this pathogen to humans (3). In Europe, chicken meat consumption was estimated to account for 20–30% of *Campylobacter*iosis cases, whereas the chicken reservoir might attribute to 50–80% of these cases (3, 4). The contamination of Campylobacter at early chicken meat production stages plays an important role in transmitting *Campylobacter* from farm to fork. The prevalence of *Campylobacter* on the farm and during process in the slaughterhouse can also reflect the *Campylobacter* contamination in meat products (3, 5).

*Campylobacter* can appear in broiler chicken as early as 14-day age at rearing with a low percentage and increase to a high contamination level at the end of grow-out period (6). During the broiler chicken commercial production, flocks usually consist of 10,000–30,000 chickens per house (3). *Campylobacter* rarely transmits vertically from parents to chicks, whereas flocks at commercial production have a high risk of *Campylobacter* amplification and rapid spread due to the intensive production model (6). The slaughter process is one of the most important factors causing *Campylobacter* cross-contamination in chicken. During the slaughter process, the intestinal content will inevitably contaminate the broiler carcass and the slaughtering environment, which will further introduce *Campylobacter* to chicken meat (7).

In China, random sampling is the most common method to evaluate the prevalence of *Campylobacter* in the poultry processing line (5). However, the information provided by these prevalence studies was limited, that the potential transmission routine during broiler chicken production could only be primarily investigated. Our previous study with a “label-tracking method” in slaughterhouse showed the evisceration had the highest *Campylobacter* positive rate of 97.5% and a contamination load of 2.80 ± 2.52 LogCFU/100 cm^2^ (5). By now, limited numbers of studies have conducted the investigation on the trackable contamination of *Campylobacter* from rearing to slaughtering, which can provide more information for the risk assessment decisions.

Virulence factors related to *Campylobacter* pathogenesis and stress response including the adhesion, invasion, chemotaxis, motility, toxin-activity, immune-evasion, iron-uptake, and secretion system (7). Genes associated to virulence have already been detected by several studies to evaluate the potential risk of *Campylobacter* isolates to the food safety and public health including *flaA* for flagellin protein FlaA, outer membrane protein *cadF* for CadF for adhesion, *cdtA, cdtB*, and *cdtC* for cytolethal distending toxin (CDT) subunits, *cheY* for chemotaxis-related response regulator CheY, *iamA* for invasion-associated protein *iamA* and *virB*11 for *Campylobacter* invasion located on plasmid (8–10). Recently, the Type VI secretion system (T6SS) have been known to be important for *Campylobacter* stress survival and pathogenesis, which the *hcp* gene encoding hemolysin coregulated protein was regarded as a key component for evaluating the pathogenesis and stress resistance of *Campylobacter* isolates from different sources (8, 11, 12).

The multidrug-resistant (MDR) *Campylobacter* have been frequently reported from clinic and poultry meat production. *Campylobacter* has been reported to resistant to several antibiotics including fluoroquinolones, tetracyclines, macrolides, aminoglycosides and β-lactams (13). The persistence of MDR *Campylobacter* during the early poultry production stage was corresponding to the uncontrolled antibiotic use during the production, which provides a selection pressure for MDR *Campylobacter* spreading in the production chain (14).

This study aimed to investigate the transmission of *Campylobacter* from broiler rearing period to slaughtering stages by applying a tracking method. The isolates from tracking samples were selected for whole genome sequence analysis and characterized by MLST and the presence of antibiotic resistance genes and virulence factors.

## Materials and Methods

### Experimental Design and Sample Collection

To investigate the transmission of *Campylobacter* from the broiler rearing to slaughter production, this study conducted the sampling from the same selected broiler chickens from rearing to slaughtering by a tracking method. The sampling were conducted according to the advice from a chicken slaughterhouse in Eastern China. The broiler farm was selected as the broiler supplier for the slaughterhouse. The slaughterhouse normally slaughtered 10,000–15,000 broilers per day. In total, three broiler flocks were chosen with relatively small scales of 5,000–10,000 boilers per flock. At each flock, 50 broiler chickens were randomly selected and labeled by plastic vervel tag with numbers at 31-day age. Cloacal samples of each labeled broiler chicken were taken at 31-day age, 37-day age, and at the age when chicken entering slaughterhouse (41–44-day age). At the slaughterhouse, chicken carcass were sampled at four slaughtering stages according to the Hazard Analysis and Critical Control Point including dehairing, evisceration, cooling, and partition (5). During slaughtering, chicken carcasses were hanged for dehairing and evisceration. At the cooling stage, chickens carcasses went through a water pre-cooling tank containing 50 mg/kg sodium hypochlorite with a temperature of 10°C before hanging for partition. Labeled chicken carcasses were tracked as much as possible at four sampling steps during the slaughter process. In addition, random sampling was also conducted at each slaughter sampling stage to overcome the limited sampling size of tracking samples, and investigate the prevalence of *Campylobacter* during the broiler chicken slaughter process.

In total, 1,534 samples were taken from chicken rearing stage, slaughtering stages, and their relative environments. The detail sampling size for each stage was listed in Table 1. Samples were obtained from the slaughterhouse at a medium-scale slaughterhouse in Eastern China during August to October 2018. Samples were collected as previously described (5). In brief, each cloacal sample was collected by a sterilized cotton stick and stored in Cary-Blair transport medium. At each slaughtering step, wiping samples were collected by phosphate buffer (PBS, pH 7.2) immersed sterilized cotton balls. The whole chicken carcasses surface were swabbed after dehairing, while half exterior surface and half interior surface of the chicken carcasses were swabbed after evisceration, cooling and partition, which the sampling area were ~250 cm^2^ for each sample (5). Environmental samples at the rearing stage included feed, water, floor, sole, net, bedding, and stool. Feed, water, bedding and stool samples were directly picked up from chicken flocks, and floor, sole and net samples were collected by the surface wiping method. Environmental samples at the slaughtering stages were all collected by wiping the process related machine surface of 250 cm^2^. Each collected sample was sealed in a sterile homogeneous bag and directly transported to the laboratory for further treatment.

**Table 1:** Prevalence of *Campylobacter* from broiler chicken during rearing and slaughtering.

**Source**	**Sample size**	**No. of positive**	**Prevalence (%)**	***C. jejuni***	***C. coli***
**Rearing period**					
31-day age	150	59	39.3	46	26
37-day age	150	90	60	74	38
**Slaughter operation**					
Entrance (41–44 day age)	145	60	41.4	38	22
Dehairing	201	26	12.9	3	23
Evisceration	191	102	53.4	37	69
Cooling	183	27	14.8	12	16
Partition	176	24	13.6	8	18
Total	1196	388	32.4	218	212

### Identification of *Campylobacter* spp.

For cloacal samples, each cotton stick was immersed in 1 ml PBS for 20 min. For chicken carcass wiping samples, PBS in cotton balls of each sample was squeezed to an Eppendorf tube. *Campylobacter* from both cloacal samples and carcass wiping samples was identified as previously described Huang et al. (15). Briefly, PBS rinsing solution was serially diluted in saline, and 100 μl of each dilution serial was spread on *Campylobacter* selective blood free agar mCCDA (modified CCDA, Preston, Oxoid, UK) plate with antibiotics (16). All plates were incubated at 42°C for 48 h under microaerobic condition with 10% CO_2_, 5% O_2_, 85% N_2_. Four or five of presumptive *Campylobacter* colonies from each mCCDA plate were further identified as *Campylobacter jejuni* or *Campylobacter coli* by multiplex PCR. MALDI-TOF analysis was conducted to isolates with identical *Campylobacter* colony morphology, but could not be identified by multiplex PCR. The multiplex PCR targeted to genes including 16S rRNA for *Campylobacter* genus, *mapA* for *C. jejuni* and *ceuE* for *C. coli*. Primers for this multiplex PCR were listed in Table S1. The program of the multiplex PCR was performed with an initial denaturation at 95°C for 10 min, followed by 30 cycles of denaturation at 95°C for 30 s, annealing at 59°C for 90 s, and elongation at 72°C for 60 s, and a final extension at 72°C for 10 min.

### Genomic Characterization of *Campylobacter* From Tracking Samples

From the tracking broiler samples, 11 samples were able to isolate *Campylobacter* from rearing period to slaughtering operation. A total of 40 *Campylobacter* isolates were identified from 11 tracking broiler samples. All isolates were sequenced to investigate the contamination route of *Campylobacter* from broiler chicken rearing to slaughtering process. Genomic DNA of all chosen *Campylobacter* isolates was extracted by TIANamp Bacteria DNA Kit (Tiangen, Beijing, China) and sequenced by Illumina Hiseq 2500. Reads were assembled to contigs by SPAdes version 3.10.0 (17). Multilocus sequence typing was analyzed by PubMLST database for *Campylobacter* (https://pubmlst.org/campylobacter/). All sequenced *Campylobacter* isolates was analyzed for the core genome regions by Parsnp software, and the whole genome sequencing (WGS) data submitted to European Nucleotide Archive database with the accession number PRJEB36059 for *C. jejuni* isolates and the PRJEB36073 for *C. coli* isolates. Virulence factors were detected by blastn including *flaA, cadF, virB1*1, *cdtA, cdtB, cdtC, iamA, ciaB, cheY*, and *hcp*. Antimicrobial resistance genes of all sequenced isolates were detected by ReFinder 3.3 database (https://cge.cbs.dtu.dk/services/ResFinder/) (18).

### Statistical Analysis

The proportions of *Campylobacter* in different sampling steps were based on Chi-square analysis with SPSS statistical package (SPSS Inc., Chicago, USA). Statistical significance was set at *p* ≤ 0.05.

## Results

### Prevalence of *Campylobacter* From Broiler Chicken Rearing to Slaughtering Production

A total of 1,534 samples were collected from broiler chicken farm to the slaughterhouse, which includes 1,196 chicken cloacal samples, and 338 environment samples (Tables 1, 2). In total, 27.2% of the samples were *Campylobacter* positive. During the rearing period, a total of 300 cloacal samples from broiler chicken were collected, including 150 samples at both 31-day and 37-day age. 39.3% of chicken cloacal samples were *Campylobacter* positive at 31-day age, while the *Campylobacter* positive rate was increased to 60% at 37-day age (*p* = 0.001^**^). A total of 150 environmental samples at the rearing period, which was 1.6% of the rearing environmental samples at 31-day age and 3.2% at 37-day age, respectively (Table 2).

**Table 2:** Prevalence of *Campylobacter* in the environment during broiler rearing and slaughtering.

**Source**	**Sample size**	**No. of positive**	**Prevalence (%)**	***C. jejuni***	***C. coli***
**Rearing Environment**					
**31-day age**	**126**	**2**	**1.6**	**1**	**1**
Sole	21	1	4.7	1	–
Bedding	10	1	10	–	1
Feed	25	0	0	–	–
Water	25	0	0	–	–
Floor	15	0	0	–	–
Net	15	0	0	–	–
Stool	15	0	0	–	–
**37-day age**	**124**	**4**	**3.2**	**4**	–
Sole	19	2	10.5	2	–
Bedding	10	1	10	1	–
Stool	15	1	6.7	1	–
Feed	25	0	0	–	–
Water	25	0	0	–	–
Floor	15	0	0	–	–
Net	15	0	0	–	–
**Slaughtering Environment**					
Dehairing	22	7	31.8	3	4
Evisceration	22	7	31.8	1	7
Cooling	22	8	36.4	5	5
Partition	22	2	9.1	1	1
Total	338	30	0.89	20	19

At the slaughtering stage, 145 cloacal samples were collected before broiler entering the slaughter operation (41–44- day age broiler), which was 41.4% of *Campylobacter* positive. Compared to the cloacal samples before slaughter operation, the *Campylobacter* positive rate decreased to 12.9% at the dehairing stage (*p* = 0.000^**^), while, the percentage of *Campylobacter* positive samples had a steep rise to 53.4% during evisceration (*p* = 0.000^**^). At the cooling step, the prevalence of *Campylobacter* were decreased to 14.8% (*p* = 0.000^**^) and continuously kept at a low level (13.6%) during partition (*p* = 0.879). In slaughtering environment, the *Campylobacter* contamination from the machine surface were similar at dehairing (31.8%), evisceration (31.8%), and cooling (36.3%), and dramatically decreased at partition (9.1%) (Table 2).

A total of 233 *C. jejuni* isolates and 231 *C. coli* isolates were identified from the collected samples from the rearing period to the slaughtering process. At the rearing stage, a total of 120 *C. jejuni* isolates and 64 *C. coli* isolates were identified from broiler chicken cloacal samples. Before entering the slaughterhouse (41–44 day age), 30 *C. jejuni* isolates and 22 *C. coli* isolates were identified from the cloacal samples (Table 1). One hundred twenty-six *C. coli* isolates and 60 *C. jejuni* were identified from swabbing samples collected during the slaughtering process (Table 1).

### Distribution of *Campylobacter* spp. From the Rearing Period to the Slaughtering Process

In total, 59 tagged broiler chickens with tag numbers were able to identify *Campylobacter* during rearing and/or slaughtering, which provide a primary distribution data of *Campylobacter* spp. for early chicken meat production (Table S2). *Campylobacter jejuni* were predominant during the rearing period, while *C. coli* were more frequently detected during the slaughter process. During the rearing period, no *C. coli* were detected at 31-day age and only four samples were identified with *C. coli* at 37-day age. Before entering the slaughter process, the distribution of *C. jejuni* and *C. coli* in labeled broiler chicken cloacal samples were at the same level, which were 25 and 22 positive samples, respectively. During slaughtering, *C. jejuni* was only detected in two samples from the dehairing step, and 11 samples from the evisceration step. On the other hand, *C. coli* were more prevalently observed from 11 of dehairing samples and 32 of evisceration samples. Both cooling and partition stage showed a sharp decrease of *Campylobacter* prevalence, which only one *C. jejuni* and one *C. coli* were identified from the cooling samples and one *C. coli* from the partition samples. In addition, one sample from 37-day age rearing stage and six samples from the evisceration steps during slaughter were contaminated by both *C. jejuni* and *C. coli*.

### Genomic Characterization of *Campylobacter* From Tracking Broiler Samples

**Figure 1:**
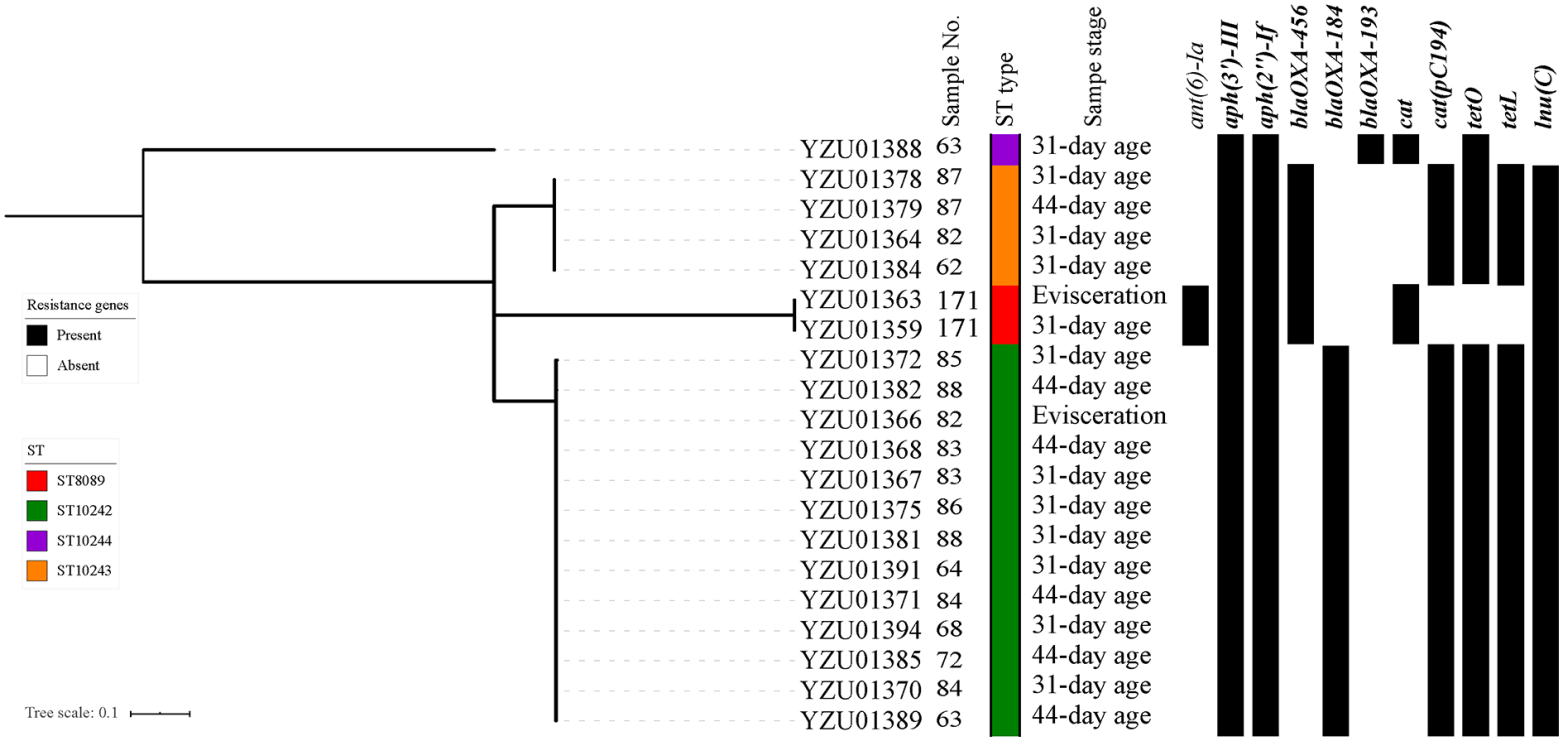
Phylogenetic tree based on core genome and drug resistance genes of *Campylobacter jejuni*. The analysis included 20 *C. jejuni* isolates from the tagged broiler sample from rearing period to the slaughter operation. The antimicrobial resistant genes were listed according to the WGS data.

**Figure 2:**
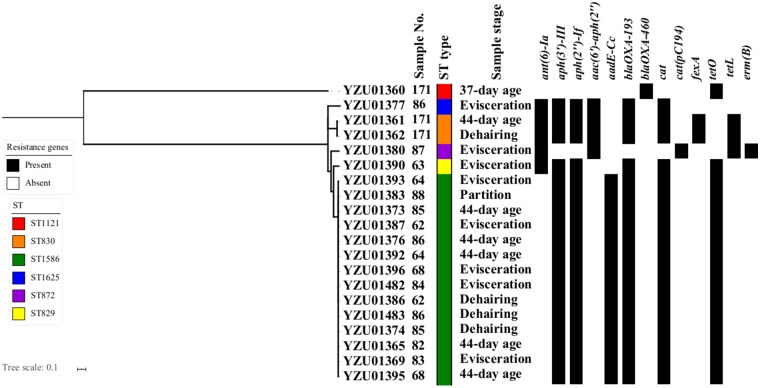
Phylogenetic tree based on core genome and drug resistance genes of *Campylobacter coli*. The analysis included 20 *C. coli* isolates from the tagged broiler sample from rearing period to the slaughter operation. The antimicrobial resistant genes were listed according to the WGS data.

During the sampling process, 11 labeled samples were able to identify *Campylobacter* from both rearing period (31-day and/or 37-day age chicken) and slaughtering stages. A total of 40 *Campylobacter* isolates were identified including 20 *C. jejuni* isolates and 20 *C. coli* isolates (Table S3). All 40 *Campylobacter*isolates were whole genomic sequenced and conducted MLST and core genomic analysis to evaluate the similarity of *Campylobacter* isolates during the early broiler chicken production. In total, four ST types were observed in sequenced *C. jejuni* isolates from labeled samples including ST8089, ST10242, ST10244, and ST10243. The core genomic analysis divided *C. jejuni* isolates into four clusters with a predominant cluster of isolates with ST10242 mainly from cloacal samples at both rearing stages and before entering the slaughter process (Figure 1). Two labeled samples (No. 87 and 171) were able to identify *C. jejuni* from the same STs during rearing and slaughtering. For *C. coli* isolates, six different ST types were observed including ST1121, ST830, ST1568, ST1625, ST872, and ST829 (Figure 2). Fourteen *C. coli* isolates were grouped to ST1568, which were from nine labeled broiler chicken samples at slaughtering stages including cloacal samples before entering the slaughtering process (41–44-day age) and swabbing samples at dehairing, partition, and evisceration steps during slaughter (Figure 2). In addition, ST1568 isolates were detected at different slaughter steps in four labeled samples including sample No. 62, 64, 68, and 86. Meanwhile, ST830 were observed form labeled chicken No. 171 from cloacal sample before entering the slaughter process line and dehairing step (Figure 2). The detection of the same ST types of *C. jejuni* and *C. coli* indicated the transmission of the same ST types during the slaughtering process.

### Virulence Factors and Antibiotic Resistance Genes in *Campylobacter* spp.

Virulence factors were detected in all sequenced *C. jejuni* and *C. coli* isolates from tracking broiler chicken samples. All *C. jejuni* isolates contained eight virulence factors including *flaA, cadF, cdtA, cdtB, cdtC, iamA, ciaB, cheY* (Table 3). Three different virulent haplotypes were observed in the *C. coli* isolates, which one isolate belonged to ST1625 only containing *cheY*, two isolates belonged to ST830 containing *flaA, cheY* and *virB11*, and the rest of sequenced *C. coli* isolates (*n* = 17) containing *flaA* and *cheY* (Table 3).

**Table 3:** Virulotyping of *Campylobacter* isolates with different STs.

**Species**	**Virulotype**	**Number of strains**
***Campylobacter jejuni***		
	*flaA-cadF-cdtA-cdtB-cdtC-iamA-ciaB-cheY*	*n* = 2 (ST8089); *n* = 13 (ST10242); *n* = 1 (ST10244); *n* = 4 (ST10243)
***Campylobacter coli***		
	*cheY*	*n* = 1(ST1625)
	*flaA-cheY*	*n* = 1 (ST1121); *n* = 1 (ST872); *n* = 1 (ST829); *n* = 14 (ST1586)
	*flaA-cheY-virB11*	*n* = 2 (ST830)

In total, eleven antibiotic resistance genes (ARGs) were detected from 20 sequenced *C. jejuni* isolates (Figure 1) and thirteen ARGs were detected from 20 sequenced *C. coli* isolates (Figure 2). All *C. jejuni* isolates displayed ARGs resistant to four different antibiotic groups, and all *C. coli* isolates contained ARGs resistant to at least two different antibiotics. The presence of ARGs in both *C. jejuni* and *C. coli* were correlated to the ST types, which each ST type contained a distinct antibiotic resistance gene type (Figures 1, 2). All *C. jejuni* isolates and 18 out of 20 *C. coli* isolates contained aminoglycoside-resistant genes *aph(3')-III, aph(2”)-If*, and *ant(6)-Ia* was presented in two *C. jejuni* isolates and five *C. coli* isolates, and *aac(6')-aph(2”)* and *aadE-Cc* were only present in *C. coli* isolates. β-lactam resistant gene in *C. jejuni* and *C. coli* were diverse, which *bla*_OXA−456_ and *bla*_OXA−184_ were only present in *C. jejuni*, and *bla*_OXA−460_ were only present in one *C. coli* isolates. *bla*_OXA−193_ were observed in one *C. jejuni* isolate and 17 out of 19 *C. coli* isolates. The distribution of chlorophenol resistant genes were also distinct, which the *cat* gene was observed in three *C. jejuni* isolates and 17 out of 20 *C. coli* isolates, while *cat(pC194)* was observed in 17 *C. jejuni* isolates and one *C. coli* isolate, and *fexA* genes were presented in two *C. coli* isolates. Tetracycline resistant genes *tetO* were detected in 18 *C. jejuni* isolates and 16 *C. coli* isolates, while *tetL* were detected in 17 of *C. jejuni* isolates and three *C. coli* isolates. Macrolide resistant gene *lnu(C)* was observed in 19 *C. jejuni* isolates, while *emr(B)* were observed in one *C. coli* isolates.

## Discussion

This study applied a tracking method to detect the prevalence of *Campylobacter* from the broiler chicken rearing period and the slaughter process. At the broiler rearing stage, 150 broilers were tagged by a specific number and tracked for *Campylobacter* positive by the cloacal sample at 31-day age and 37-day age and before slaughtering. During the rearing period, the *Campylobacter* positive rate was increased from 39.3 to 60.0%, while was decreased to 41.4% at 41–44- day age before entering the slaughter operation (Table 1). Before slaughtering, feed withdrawal is an important process to ensure the broiler chicken gastrointestinal system as clean as possible, which could increase the difficulty of cloacal samples due to the lack of intestinal contents (19). In Europe, the prevalence of *Campylobacter* was diverse among different countries, with the high prevalence observed in UK (87.5%) and the Netherland (80%), while the prevalence in Denmark, Germany and France was similar to this study with 40–50% (20, 21). The low prevalence of *Campylobacter* was reported in Norway (18%) and Italy (17.38%) (20, 22). In Asia, the prevalence of *Campylobacter* was 26.3% in Thailand and 45% in Japan (23, 24). The environment sample during slaughtering showed a very low *Campylobacter* contamination.

At the slaughter process, the positive percentage of *Campylobacter* changed dramatically during different processing steps. The highest prevalence of *Campylobacter* contamination was observed at the evisceration step due to the expose of intestinal contents. The cooling step is crucial for eliminating *Campylobacter* on chicken carcasses, that the *Campylobacter* positive rate decreased from 53.4% during evisceration to 14.75% after cooling. During cooling, the slaughterhouse applied sodium hypochlorite combined with low temperature, which contributed to the reduction of *Campylobacter*. Chlorine has been known to have bactericidal effect, and has been applied on microbial contamination during chicken processing (25). A previous study from South California also showed a significant decrease of the *Campylobacter* contamination load for 0.97 logCFU/g after the water cooling process (7, 26). In this study, the *Campylobacter* positive rate continuously kept at a low level (13.64%) during partition *Campylobacter* contamination in the environment during the slaughter process was relatively high indicating the possibility of cross contamination during slaughter.

The distribution of *Campylobacter* was different between broiler rearing period and slaughtering. The distribution of *Campylobacter* was diverse between rearing period and slaughter operation, which *C. jejuni* predominant during rearing while *C. coli* were more frequently observed during slaughtering. A previous prevalence study from China also showed that 208 *C. jejuni* isolates and 53 C, *coli* isolates were detected from 767 broiler rearing samples (27). At the slaughter operation, *C. coli* were more frequently observed compared to *C. jejuni*, which was also agreed with a previous prevalence study of *Campylobacter* in the slaughterhouse from Jiangsu province in China (13). This study applied a tracking method to track the *Campylobacter* from precise samples during the production with an initial sample size of 150 tagged broiler samples. Fifty-nine samples were able to identify *Campylobacter* at least two sampling steps during the rearing period and the slaughtering process. However, this method also had a drawback of the loss of sample tag during the slaughter process, which only influenced the sample sizes for the further analysis. The drawback were remedied by an additional random sampling during the process in our study.

The whole genomic analysis was performed on 40 *Campylobacter* isolates from 11 labeled samples to track the variation of *Campylobacter* at both the rearing stage and during the slaughter operation. The MLST and core genome analysis showed the cross-contamination of *Campylobacter* from the rearing period to the slaughter operation. At the rearing stage, *C. jejuni* ST10244 and ST10243 and *C. coli* ST830 and ST1586 were isolated from the same tagged broiler chicken samples at different production stages indicating that the rearing stage contamination of *Campylobacter* could influence the *Campylobacter* contamination in the downstream of food production chain. *C. jejuni* ST10242 was predominant at the broiler rearing period. The prevalence of *C. coli* ST1586 at the slaughter operation instead of *C. jejuni* ST10242 from rearing period indicating that cross-contamination during slaughter operation could be a main contribution of *Campylobacter* contamination in chicken meat products. *C. jejuni* ST 8089 was reported to be predominant during chicken slaughtering process from a previous Chinese study (13). *Campylobacter coli* ST1121 and ST1625 were only reported from animals (16, 28, 29), while *C. coli* ST830, ST1586, and ST829 was shared between poultry and humans indicating a potential threat to the public health by transmitting to humans through the production chain (24, 30).

We further analyzed the presence of virulence factors in both *C. jejuni* and *C. coli*, and observed a distinct different between the two species. All sequenced *C. jejuni* contained only one virulence gene pattern with the presence of eight out of nine detected virulent factor genes including *flaA, cadF, cdtA, cdtB, cdtC, iamA, ciaB, cheY*, which involved pathogenesis of motility, adhesion, invasion, toxin production, and chemotaxis. On the other hand, *C. coli* contained three different virulence gene patterns with the presence of only three virulent determinant genes including *flaA, cheY*, and *virB*11. A previous Canadian study demonstrated virulence factors in *C. jejuni* isolates from poultry meat relating to toxin production (*cdtA, cdtB, cdtC*), cell adhension (*cadF*) and invasion (*ciaB, iam*) (31). Meanwhile, A recent study of *C. coli* from duck sources in Korea reported the predominant of *flaA, cadF, ceuE*, and *cdtA*, while *iamA, virB11* and *hcp* were also sporadically observed (30). Previous studies also demonstrated that *C. jejuni* carried more virulent-related genes compared to *C. coli*, which might contribute to the survival and colonization of *C. jejuni* in the poultry intestines (16, 32).

In this study, all sequenced *Campylobacter* isolates were analyzed for the presence of ARGs. In addition, the antibiotic resistant genotypes were well-corresponding to the ST types (Figures 1, 2). The aminoglycoside-resistant genes *ant(6)-Ia, aph(3')-III, aph(2”)-If* in *C. jejuni* and *C. coli* were frequently reported from other studies, which were related to the resistance to aminoglycoside (33, 34). All isolates contained the *catA* gene were associated with the resistance to chloramphenicol, which is relatively high compared to other studies. The *cat* gene had a prevalent of 14.3% in *C. jejuni* from broiler in Shanghai, China in 2016 (35). The WGS study of *C. jejuni* from the US revealed that two of 114 isolates carrying the *catA* gene (33). The *fexA* gene were related to phenicol exporter was presented in two *C. coli* isolates ST830 (33, 36). The tetracycline resistance gene *tetO* was reported from the previous study which were located on plasmid causing the spread of *tetO* in *C. jejuni* (33, 37). In addition, *tetO* was also observed in *C. jejuni* isolates from wild bird and humans (22). On the other hand, the *tetL* gene were only reported in *Campylobacter* spp. from patient in Taiwan (38). Nineteen out of twenty *C. jejuni* isolates contained the *lnuC* gene, which was associated with the resistance to lincomycin but susceptible to clindamycin and erythromycin. The *lnuC* gene in *Campylobacter* were firstly reported in the US, which two *C. coli* isolates from human were *lnuC* positive, and was rarely reported in livestock and meat (14, 33). The *lnuC* gene has not reported in *Campyloabcter* spp. in China yet. One of *C. coli* isolate belonging to ST872 contained the *emr(B)* gene associating with macrolide resistance in China. The *emr(B)* gene was located on the multidrug resistance genomic islands, which were most frequently observed in *C. coli* in China from both clinic and poultry isolates (39, 40).

## Conclusion

A previous study by the Swedish *Campylobacter* program showed that *Campylobacter* contamination at the farm level could increase *Campylobacter* contamination at the slaughter level (41, 42). This study investigated the overall prevalence of *Campylobacter* from the broiler chicken rearing period to the slaughter process and characterized *Campylobacter* isolates by WGS analysis. The tracking sampling method provided evidence of *Campylobacter* species diversity from the broiler rearing period to the slaughtering process. We observed the predominant of *C. jejuni* during broiler rearing period and the predominant of *C. coli* during slaughtering operation, and provided evidence of *Campylobacter* transmission from the same label-tagged broiler samples during rearing period to the slaughter operation. In addition, the virulent factor in *C. jejuni* isolates and *C. coli* isolates showed a distinct difference. Meanwhile, multidrug-resistant patterns were observed in all sequenced isolates indicating a potential risk of transmission in the broiler meat production chain. Further studies would be required to analyze risk factors for *Campylobacter* contamination during the early stage broiler production, and the potential risk of the transmission of *Campylobacter* contamination to the final meat products.

## Data Availability Statement

The WGS data has been published in ENA database. Requests to access the datasets should be directed to Yuanyue Tang, tangyy@yzu.edu.cn.

## Ethics Statement

This study was carried out in accordance with the principles of the Basel Declaration and recommendations of the institutional administrative committee and ethics committee of laboratory animals, Animal Welfare and Ethics Committees of Yangzhou University. The protocol was approved by the Animal Welfare and Ethics Committees of Yangzhou University.

## Author Contributions

YT, QJ, LK, and JH contributed to the conception and design of this study. YT, QJ, ZW, YY, and HT were responsible for the acquisition of the data analyzed in this study. YT, QJ, FR, JH, and XJ were involved in the analysis and interpretation associated with this work. All the authors were involved in manuscript revisions and final approval of the version to be published.

## Conflict of Interest

LK was employed by the company Heyi Food Co. Ltd. The remaining authors declare that the research was conducted in the absence of any commercial or financial relationships that could be construed as a potential conflict of interest.
